# Fracture Risk and Health Profiles Differ According to Relationship Status: Findings from the Hertfordshire Cohort Study

**DOI:** 10.1007/s00223-024-01194-4

**Published:** 2024-03-18

**Authors:** Leo D. Westbury, Camille Pearse, Gregorio Bevilacqua, Nicholas R. Fuggle, Kate A. Ward, Cyrus Cooper, Elaine M. Dennison

**Affiliations:** 1 MRC Lifecourse Epidemiology Centre, University of Southampton, Southampton, UK; 2 The Alan Turing Institute, London, UK; 3 NIHR Southampton Biomedical Research Centre, University of Southampton and University Hospital Southampton NHS Foundation Trust, Southampton, UK; 4 NIHR Oxford Biomedical Research Centre, University of Oxford, Oxford, UK; 5 Victoria University of Wellington, Wellington, New Zealand

**Keywords:** Relationship status, Fracture, Lifestyle, Socioeconomic, Osteoporosis, Epidemiology

## Abstract

Registry studies have suggested associations between relationship status and fracture risk. We considered associations between relationship status and incident fracture in the Hertfordshire Cohort Study, comprising community-dwelling older adults, and explored associations between socioeconomic and lifestyle factors with relationship status. 2997 participants completed a baseline questionnaire (1998–2004) and clinic visit. Participants were followed up until December 2018 using Hospital Episode Statistics, which report clinical outcomes using codes from the 10th revision of the International Classification of Diseases (ICD-10); these codes were used to ascertain incident fractures. Relationship status (not currently married/cohabiting vs currently married/cohabiting) at baseline was examined in relation to incident fracture using Cox regression. Associations between baseline characteristics and relationship status were examined using logistic regression. Mean baseline age was 66.2 years. 80% were married/cohabiting at baseline; 15% had an incident fracture (mean (SD) follow-up duration: 14.4 (4.5) years). The following were related to greater likelihood of not being married/cohabiting: older age (women only); higher BMI (women only); current smoking; high alcohol consumption (men only); poorer diet quality (men only); lower physical activity; leaving school before age 15 (women only); and not owning one’s home. Those not married/cohabiting had greater risk of incident fracture compared to those who were (age-adjusted hazard ratios (95% CI) 1.58 (1.06, 2.38) among men, 1.35 (1.06, 1.72) among women); associations were attenuated after accounting for the above factors associated with relationship status in the corresponding sex. This suggests that differences in health profiles and lifestyle according to relationship status may explain the association between relationship status and fracture risk.

**Supplementary Information:**

The online version contains supplementary material available at 10.1007/s00223-024-01194-4.

## Introduction

Osteoporotic fractures are a cause of significant mortality and morbidity, with huge personal and societal costs. For example, among Caucasians, approximately one in two women and one in five men over the age of 50 years will sustain an osteoporotic fracture at some point in their remaining lifetime [1], and the economic burden of osteoporotic fractures in the European Union was estimated to be €37 billion in 2010 [2]. Relationship status has previously been associated with health outcomes including premature mortality [3], cardiovascular disease [4] and falls [5]; some studies have also suggested that relationship status is associated with hip fracture [6–9].

Anthropometric factors such as BMI, and lifestyle factors such as smoking and excessive alcohol consumption are widely established risk factors for osteoporotic fractures [10]. There is evidence that single people may have poorer health behaviours than those in relationships [11, 12], making it plausible that relationship status may be related to fracture risk through the established association between relationship status and lifestyle. However, few studies that have contemporaneous detailed information on lifestyle factors have explored the association between relationship status and fracture risk while accounting for these factors, and also examined whether these factors differ according to relationships status. To address this, we considered these associations in the Hertfordshire Cohort Study, a cohort of community-dwelling older adults for whom detailed lifestyle information was available.

## Methods

### The Hertfordshire Cohort Study

The Hertfordshire Cohort Study (HCS) comprises 2997 men and women born in Hertfordshire between 1931 and 1939, and who still lived there in 1998–2004 when they completed a home interview and clinic visit for a health assessment. The HCS had ethical approval from the Hertfordshire and Bedfordshire Local Research Ethics Committee and all participants provided written informed consent for the investigations they underwent in clinic and for researchers to access their medical records in the future. Further details of HCS have been described previously [13, 14].

### Ascertainment of Participant Information in 1998–2004

Marital status (out of the options ‘single’, ‘married’, ‘divorced or separated’, ‘widowed’, or ‘cohabiting’) was reported by participants and then dichotomised for all analyses into not currently married/cohabiting (comprising participants who were single, divorced, separated or widowed) and currently married/cohabiting. Smoking status, alcohol consumption and physical activity (Dallosso questionnaire [15]) were ascertained by a clinician-administered questionnaire. Participants completed a food-frequency questionnaire from which a prudent diet score was derived with higher scores reflecting healthier diets.

Social class was ascertained from current or most recent full-time occupation for all men and among women who never married, and from husband’s occupation for ever-married women. Social class was coded from the 1990 OPCS standard occupational classification (SOC90) unit group for occupation [16]. Socioeconomic characteristics ascertained included: age left education (dichotomised as < 15 years vs. ≥ 15 years); social class (manual vs non-manual) and housing tenure (owned/mortgaged vs not).

At the baseline clinic, measurements were made of height (Harpenden pocket stadiometer, Chasmors Ltd, London, UK) and weight (SECA floor scale, Chasmors Ltd, London, UK) which were used to derive body mass index (BMI).

### Ascertainment of Incident Fractures

Hospital Episode Statistics (HES) data were used to identify incident fractures; data on mortality were also available. Permission to access these data from participants from HCS baseline to 31/12/2018 was provided by NHS Digital and the Ethics and Confidentiality Committee of the National Information Governance Board. The process of linking the HCS cohort with HES data has been explained in a previous publication [17]. The extracted HES data for each participant included details regarding hospital admissions, including diagnoses categorized using codes from the 10th revision of the International Classification of Diseases (ICD-10). Adverse health events considered included fractures of any kind, as well as specifically hip fractures. These fracture events were identified using the ICD-10 codes outlined in eTable 1 (Online Resource). These ICD-10 codes for identification of any fractures [18, 19] and hip fractures [20–22] have been used in previous studies.

### Statistical Methods

Participant characteristics were described using summary statistics. Cross-sectional associations between baseline characteristics and relationship status (not married/cohabiting vs. married/cohabiting) were examined using sex-stratified logistic regression. Baseline relationship status was examined in relation to incident fracture outcomes (any fracture and hip fracture) using sex-stratified time-to-first-event Cox regression with adjustment for age and with death as a censoring event. Fully adjusted sex-stratified Cox models were then implemented where each model was additionally adjusted for the baseline characteristics which were significantly associated with relationship status in the corresponding sex. Analyses were conducted using Stata, release 17.0; *P* < 0.05 was regarded as statistically significant.

For sensitivity analyses, competing risk analyses were performed for the fracture outcomes using the Fine-Gray sub-distribution hazards model with death as a competing event [23].

## Results

### Descriptive Statistics

Participant characteristics of the analysis sample are illustrated in Table 1. Mean (SD) age of participants at baseline was 65.7 (2.9) and 66.6 (2.7) years among men and women, respectively. Overall, 14% of men and 27% of women were not married or cohabiting at baseline (were either single, divorced, separated, or widowed). Mean (SD) follow-up duration until the ‘any fracture’ event or until participants were censored was 14.4 (4.5) years. Over the follow-up period, 22% of women sustained a fracture, including 5% who sustained a hip fracture; corresponding figures in men were 9% and 2%. Of those who experienced fractures during follow-up, around 55% of men and women had fractures with the following ICD-10 codes which are common locations of major osteoporotic fractures: S32 (fracture of lumbar spine and pelvis); S42 (fracture of shoulder and upper arm); S52 (fracture of forearm); S62 (fracture at wrist and hand level); S72 (fracture of femur); or T08 (fracture of spine, level unspecified).

**Table 1:** Participant characteristics

Participant characteristic [mean (SD), median (lower quartile, upper quartile), or %]	Men (*n* = 1579)	Women (*n* = 1418)
*Characteristics at baseline (1998–2004)*		
Age (years)	65.7 (2.9)	66.6 (2.7)
Height (cm)	174.2 (6.5)	160.8 (5.9)
Weight (kg)	82.4 (12.7)	71.4 (13.4)
BMI (kg/m^2^)	27.2 (3.8)	27.6 (4.9)
Current smoking	15%	10%
High alcohol intake (units per week: > 21 men, > 14 women)	22%	5%
Prudent diet score	− 0.6 (2.1)	0.7 (1.7)
Dallosso physical activity score	60.9 (15.3)	59.0 (15.7)
Left school before age 15	19%	17%
Social class (manual)	59%	58%
Home ownership (not owned or mortgaged)	19%	22%
Relationship status		
Single	5%	4%
Married	84%	71%
Divorced or separated	6%	7%
Widowed	4%	17%
Cohabiting	2%	2%
Relationship status (not married or cohabiting)^a^	14%	27%
*Events during follow-up (ever had)*		
Any fracture	9%	22%
Hip fracture	2%	5%

Follow-up period lasted from baseline (1998–2004) until 31st December 2018

^a^Participants who reported their baseline relationship status as ‘single’, ‘divorced or separated’, or ‘widowed’ were regarded as not married or cohabiting at baseline

### Associations Between Baseline Participant Characteristics and Relationship Status

Univariate cross-sectional associations between baseline participant characteristics and relationship status are presented in Table 2. The following characteristics were related to greater likelihood of not being married or cohabiting at baseline: older age (women only); higher BMI (women only); current smoking; high alcohol consumption (men only); poorer diet quality (men only); lower physical activity; leaving school before age 15 (women only); and not owner-occupying one’s home.

**Table 2:** Univariate odds ratios for not being married or cohabiting at baseline according to baseline participant characteristics

Participant characteristic	Men		Women	
	Odds ratio (95% CI)	*P*-value	Odds ratio (95% CI)	*P*-value
Age (years)	1.01 (0.96, 1.06)	0.790	**1.09 (1.05, 1.14)**	** < 0.001**
Height (cm)	0.99 (0.97, 1.01)	0.371	0.99 (0.97, 1.01)	0.509
BMI (kg/m^2^)	1.00 (0.96, 1.04)	0.934	**1.03 (1.01, 1.05)**	**0.016**
Current smoker	**1.75 (1.23, 2.48)**	**0.002**	**1.70 (1.18, 2.45)**	**0.004**
High alcohol intake	**1.45 (1.05, 2.00)**	**0.023**	0.97 (0.56, 1.68)	0.908
Prudent diet score	**0.91 (0.85, 0.98)**	**0.008**	0.95 (0.89, 1.02)	0.164
Dallosso physical activity score	**0.99 (0.98, 1.00)**	**0.044**	**0.99 (0.98, 1.00)**	**0.003**
Left school before age 15	1.25 (0.89, 1.76)	0.198	**1.37 (1.02, 1.85)**	**0.037**
Social class (manual)	1.29 (0.96,1.74)	0.090	0.91 (0.72, 1.15)	0.428
Home ownership (not owned or mortgaged)	**3.65 (2.69, 4.94)**	** < 0.001**	**2.25 (1.73, 2.94)**	** < 0.001**

Odds ratios correspond to unit increases in the characteristic or the presence versus absence of the characteristic

High alcohol intake: > 21 units per week for men and > 14 units per week for women

Statistically significant associations (*P* < 0.05) are highlighted in bold

### Associations between Relationship Status and Incident Fracture Outcomes

Associations between relationship status and risk of incident fracture are presented in Table 3. Among men and women, those who were not married or cohabiting at baseline had greater risk of incident fracture compared to those who were after adjustment for age (hazard ratios (95% CI) 1.58 (1.06, 2.38) among men, 1.35 (1.06, 1.72) among women). However, these associations were attenuated among both sexes (*P* = 0.078 among men, *P* = 0.079 among women) after adjustment for the participant characteristics that were associated with relationship status in Table 2.

**Table 3:** Hazard ratios for incident fracture outcomes for participants who were not married or cohabiting at baseline compared to those who were

Sex	Model	Any fracture		Hip fracture	
		Hazard ratio (95% CI)	*P*-value	Hazard ratio (95% CI)	*P*-value
Men	Age-adjusted	**1.58 (1.06, 2.38)**	**0.027**	2.07 (0.98, 4.39)	0.057
	Fully adjusted	1.46 (0.96, 2.23)	0.078	1.95 (0.89, 4.28)	0.095
Women	Age-adjusted	**1.35 (1.06, 1.72)**	**0.014**	1.27 (0.78, 2.06)	0.333
	Fully adjusted	1.25 (0.97, 1.60)	0.079	1.19 (0.73, 1.95)	0.487

Time-to-first-event Cox regression was used; death was regarded as a censoring event. Fully adjusted models for men accounted for age, smoking status, alcohol consumption, diet quality, physical activity and housing tenure; fully adjusted models for women accounted for age, BMI, smoking status, physical activity, age left education and housing tenure

Statistically significant associations (*P* < 0.05) are highlighted in bold

Associations between relationship status and risk of incident hip fracture were weak among women after adjustment for age and in fully adjusted analysis. Stronger associations were observed among men between not being married or cohabiting and increased risk of hip fracture (2.07 (0.98, 4.39) after adjustment for age, *P* = 0.057); this association was attenuated in fully adjusted analysis (*P* = 0.095).

### Sensitivity Analyses

Associations were attenuated in competing risk analyses compared to associations estimated using Cox regression (eTable 2, Online Resource). However, similar effect sizes were observed from the two techniques and the direction of associations was the same.

## Discussion

In this study of community-dwelling older people, not being married or cohabiting was associated with increased risk of incident fracture in both sexes after adjustment for age. However, these associations were attenuated after adjustment for sociodemographic, anthropometric and lifestyle factors that were associated with relationship status. This suggests that differences in these factors between those who were married or cohabiting and those who were not may explain the fracture associations observed. Our results suggest that relationship status is not an independent risk factor for fracture in our cohort, but rather a marker of other factors that influence bone health and fracture risk as might be anticipated. We also found that participants who were not married or cohabiting had poorer health behaviours and lower socioeconomic position in general compared to those who were.

Previous studies have explored associations between relationship status and risk of incident fracture. Among 155,940 participants, aged 60 years and older from seven European and US cohorts, those living alone had greater risk of hip fracture compared to those who were married/cohabiting after accounting for socioeconomic and lifestyle factors and comorbidity history [6]. In a Swedish study comprising approximately 250,000 residents of Stockholm County from 1993 to 1995, age-standardised odds of hip fracture among both men and women were greater among unmarried, divorced/separated, and widowed participants compared to those who were married [7]. In another Swedish study of 4589 postmenopausal women (aged 50–81 years) during 1993–1995, those who were divorced, widowed or unmarried had a higher risk of hip fracture than married or cohabiting women (odds ratio (95% CI) 1.40 (1.06, 1.85)) after adjustment for age, employment, housing type, household income, number of persons living in household, BMI, smoking, physical activity, use of HRT and parity [8]. In a Swiss study which recorded 2454 hip fractures from 1991 to 2000 among community-dwelling adults aged 50 years and over in Geneva University Hospital, men with a hip fracture who were married were approximately three years older compared to their counterparts with a hip fracture who were not married (*P* = 0.002). In contrast, married hip fractured women were over four years younger compared to women with a hip fracture who were not married (*P* < 0.001) [9]. This was the case after adjustment for area-level income and area type (urban/rural).

These previous studies broadly support our study findings that being in a relationship is protective against fractures. However, in the Hertfordshire Cohort Study, associations with hip fracture were weak, regardless of adjustments used. This may reflect our own sample size relative to the studies described above. Despite our smaller sample size, our study may be considered valuable as it provides detailed lifestyle information according to relationship status in a cohort studied at a point in the lifecourse where fractures are becoming more common.

Sexual dimorphism is apparent in some previous literature reports. For example, the study based in Geneva University Hospital suggested that being married appeared to delay hip fracture occurrence only among men while being married was associated with earlier hip fracture occurrence among women. We hypothesize that these differences may reflect different lifestyles among men and women living alone. Certainly, in agreement with our own findings, previous studies have reported healthier lifestyles among individuals in relationships compared to those who are not. For example, among 15,001 Australian adults (mean age: 52.9 years) who participated in the annual Queensland Social Survey during the years 2005 to 2014, couples were significantly more likely to be non-smokers and meet recommendations for alcohol consumption and fruit and vegetable intake compared to those who were single [11]. Similarly, being married or cohabiting was protective against smoking and heavy drinking in a study comprising 4014 Americans, aged 60 and over, who participated in the 1999–2004 waves of the US National Health and Nutrition Examination Survey (NHANES) [12]. Unlike the Australian study, the NHANES study also explored sex-differences in associations. For example, while heavy drinking was no more common among divorced/separated and widowed women than among married/cohabiting women, heavy drinking was 2.59 (95% CI: 1.89, 3.54) times more common among divorced/separated men compared to married/cohabiting men; widowed men had a 1.67 (1.13, 2.47) times higher prevalence of heavy drinking in comparison to married/cohabiting men. Being married/cohabiting had a protective effect regarding smoking among both men and women in the NHANES study.

Our findings have several potential public health implications. Interventions to improve health behaviours could be targeted among older people who are not in relationships to address the greater prevalence of less favourable lifestyle factors in this age group. Examples of these interventions could include health education programmes, fitness and wellness classes and social support programmes. These activities may also help combat feelings of social isolation and loneliness which have been found to be related to poor health behaviours [24]. Healthcare workers could also be educated about the poorer health and increased risk of adverse health outcomes such as fracture and premature mortality among older people who are single and those who may have suffered a recent bereavement. Awareness of this and of the support available may improve patient health and wellbeing.

This study has many strengths. Fracture outcomes were recorded over approximately two decades from the time participants enrolled (1998–2004) until December 2018. The linkage of the cohort with HES data provides almost complete follow-up of fracture outcomes and avoids some biases that often affect longitudinal studies, such as healthy survivor effects. However, this study does have limitations. First, this study relied on self-reported information on physical activity and diet quality, which may be influenced by recall bias. Second, occupational social class was determined differently for married women and non-married women, where the husband’s occupational social class was used as a proxy only for married women. Third, participants were all community-dwelling, Caucasian and from the relatively wealthy county of Hertfordshire, so these findings may not apply to other people of this age range and in different settings. However, the representativeness of HCS was found to be similar to the Health Survey for England which is nationally representative [13]. Fourth, there was a lack of cohort data on psychosocial and mental health characteristics which may differ substantially between participants who were and were not in relationships. Fifth, participants only reported their current relationship status at baseline; information on the length of relationships or changes in relationship status during follow-up was not available. Finally, HES data do not provide complete coverage of fracture outcomes, for example, fractures experienced by privately funded patients attending private hospitals and those treated in other jurisdictions would not be captured.

In conclusion, we have shown that not being married or cohabiting was related to increased risk of fracture in this community-dwelling older cohort, possibly due to the greater prevalence of poor health behaviours and socioeconomic deprivation in this group. Intervention strategies to improve lifestyle factors and overall health in this group may reduce the occurrence of fractures and other adverse health outcomes.

### Supplementary Information

Below is the link to the electronic supplementary material.

Supplementary file1 (DOCX 18 kb)
