# Supplementary material for: Fracture Risk and Health Profiles Differ According to Relationship Status: Findings from the Hertfordshire Cohort Study
Source: Calcif Tissue Int. 2024 Mar 18;114(5):461–7. doi: 10.1007/s00223-024-01194-4 (PMC11060979; doi:10.1007/s00223-024-01194-4)
Supplement: Supplementary file 1 — Supplementary file1 (DOCX 18 kb) [file 223_2024_1194_MOESM1_ESM.docx]

**Online Resource**

**Article title:** Fracture risk and health profiles differ according to relationship status: findings from the Hertfordshire Cohort Study

**Journal:** Calcified Tissue International and Musculoskeletal Research

**Authors**: LD Westbury, C Pearse, G Bevilacqua, NR Fuggle, KA Ward, C Cooper, EM Dennison

**Affiliations and e-mail address for corresponding author (Elaine Dennison)**:

MRC Lifecourse Epidemiology Centre, University of Southampton, Southampton, UK

Victoria University of Wellington, Wellington, New Zealand

emd@mrc.soton.ac.uk

| **eTable 1: ICD-10 codes used to identify incident fractures in Hospital Episode Statistics data** | |
| --- | --- |
| **Fracture event** | **ICD-10 codes** |
| **Any fracture** | M80: Osteoporosis with pathological fracture  M84: Disorders of continuity of bone  S22: Fracture of rib(s), sternum and thoracic spine  S32: Fracture of lumbar spine and pelvis  S42: Fracture of shoulder and upper arm  S52: Fracture of forearm  S62: Fracture at wrist and hand level  S72: Fracture of femur  S82: Fracture of lower leg, including ankle  S92: Fracture of foot, except ankle  T02: Fractures involving multiple body regions  T08: Fracture of spine, level unspecified  T10: Fracture of upper limb, level unspecified  T12: Fracture of lower limb, level unspecified  M81: Osteoporosis without pathological fracture  M82: Osteoporosis in diseases classified elsewhere  M83: Adult osteomalacia  M90.7: Fracture of bone in neoplastic disease  S02: Fracture of skull and facial bones  S12: Fracture of neck  T90.2: Sequelae of fracture of skull and facial bones  T91.1: Sequelae of fracture of spine  T91.2: Sequelae of other fracture of thorax and pelvis  T92.1: Sequelae of fracture of arm |
| **Hip fracture** | S72.0 Fracture of neck of femur; Fracture of hip NOS  S72.1 Pertrochanteric fracture; intertrochanteric fracture; trochanteric fracture  S72.2 Subtrochanteric fracture |

| **eTable 2: Subhazard ratios for incident fracture outcomes for participants who were not married or cohabiting at baseline compared to those who were, with death as a competing event** | | | | | |  |
| --- | --- | --- | --- | --- | --- | --- |
|  |  |  |  |  |  |  |
|  |  |  |  |  |  |  |
| **Sex** | **Model** | **Any fracture** | | **Hip fracture** | |  |
|  |  | **Subhazard ratio  (95% CI)** | **P-value** | **Subhazard ratio (95% CI)** | **P-value** |  |
|  |  |  |  |  |  |  |
|  |  |  |  |  |  |  |
| **Men** | **Age-adjusted** | 1.44 (0.96,2.16) | 0.078 | 1.87 (0.88,3.94) | 0.103 |  |
|  | **Fully adjusted** | 1.40 (0.92,2.13) | 0.115 | 1.84 (0.85,3.99) | 0.120 |  |
|  |  |  |  |  |  |  |
| **Women** | **Age-adjusted** | 1.27 (0.99,1.62) | 0.059 | 1.19 (0.72,1.94) | 0.496 |  |
|  | **Fully adjusted** | 1.19 (0.93,1.54) | 0.167 | 1.14 (0.69,1.88) | 0.602 |  |
|  |  |  |  |  |  |  |
| A competing risk analysis was implemented using the Fine-Gray sub-distribution hazards model; death was regarded as a competing event | | | | | |  |
|  |  |  |  |  |  |  |
| Subhazard ratios of greater than one indicate that not being married or cohabiting was related to greater incidence of the fracture event; subhazard ratios of less than one correspond to reduced incidence | | | | | |  |
|  |  |  |  |  |  |  |
|  |  |  |  |  |  |  |
| Fully adjusted models for men accounted for age, smoking status, alcohol consumption, diet quality, physical activity and housing tenure; fully adjusted models for women accounted for age, BMI, smoking status, physical activity, age left education and housing tenure | | | | | |  |
|  |  |  |  |  |  |  |
|  |  |  |  |  |  |  |
